# Study Protocol for the Residents’ Mental Health Investigation, a Dynamic Longitudinal Study in Italy (ReMInDIt)

**DOI:** 10.3390/healthcare12101020

**Published:** 2024-05-15

**Authors:** Marta Caminiti, Michelangelo Mercogliano, Federico Cussotto, Giovanni Leonardo Briganti, Dario Genovese, Walter Priano, Giorgia Maria Ricciotti, Nicole Bonaccorso, Fabiano Grassi, Antonio Antonelli, Gloria Girolametto, Gloria Spatari, Vincenza Gianfredi, Antonella Mariniello, Mariagrazia Marisei, Giuseppa Minutolo, Angela Ancona, Valentina De Nicolò, Nausicaa Berselli, Veronica Gallinoro, Claudia Cosma, Gaia Piunno, Vincenzo Montagna, Alessandro Catalini

**Affiliations:** 1 School of Hygiene and Preventive Medicine, University of Perugia, 06100 Perugia, Italy; marta.caminiti@gmail.com; 2 Department of Public Health, University “Federico II” of Naples, 80131 Naples, Italy; 3 School of Hygiene and Preventive Medicine, Department of Sciences of Public Health and Pediatrics, University of Turin, 10126 Turin, Italy; federico.cussotto@unito.it; 4 School of Hygiene and Preventive Medicine, Department of Biomedical and Neuromotor Science, Alma Mater Studiorum, University of Bologna, Via San Giacomo 12, 40126 Bologna, Italy; giovannileonardobriganti@gmail.com; 5 Department of Health Promotion, Mother and Child Care, Internal Medicine and Medical Specialties “G. D’Alessandro” (PROMISE), University of Palermo, Via del Vespro, 133, 90127 Palermo, Italy; dario.genovese@unipa.it (D.G.); walter.priano@unipa.it (W.P.); nicole.bonaccorso@unipa.it (N.B.); 6 School of Hygiene and Preventive Medicine, Department of Biomedical Sciences and Public Health, Section of Hygiene, Preventive Medicine and Public Health, Polytechnic University of the Marche Region, 60126 Ancona, Italy; giorgina.ricciotti@gmail.com; 7 School of Hygiene and Preventive Medicine, Department of Public Health and Infectious Diseases, Sapienza University, P. A. Moro 5, 00161 Rome, Italy; fabiano.grassi@uniroma1.it (F.G.); valentina.denicolo@uniroma1.it (V.D.N.); 8 School of Hygiene and Preventive Medicine, Vita-Salute San Raffaele University, 20132 Milan, Italy; antonelli.antonio@hsr.it (A.A.); ancona.angela@hsr.it (A.A.); 9 School of Hygiene and Preventive Medicine, Department of Cardiac, Thoracic, Vascular Sciences and Public Health, University of Padua, Via Giustiniani 2, 35128 Padova, Italy; girolamettogloria@gmail.com; 10 Department of Health Sciences, University of Genoa, 16132 Genoa, Italy; 11 Department of Biomedical Sciences for Health, University of Milan, Via Pascal 36, 20133 Milan, Italy; vincenza.gianfredi@unimi.it; 12 School of Occupational Medicine, University of Florence, 50134 Florence, Italy; antonella.mariniello@unifi.it; 13 Department of Advanced Biomedical Sciences, University of Naples Federico II, 80131 Naples, Italy; mariagrazia.marisei@unina.it; 14 Food Hygiene, Nutritional Surveillance and Prevention, Department of Prevention, Provincial Healthcare Authority of Palermo, 90129 Palermo, Italy; giuseppa.minutolo@asppalermo.org; 15 Public Hygiene Service, Public Health Department, Local Health Authority of Modena, 41123 Modena, Italy; n.berselli@ausl.mo.it; 16 Medical Specialization School of Hygiene and Preventive Medicine, University of Florence, 50134 Florence, Italy; veronica.gallinoro@unifi.it (V.G.); claudia.cosma@unifi.it (C.C.); 17 Department of Biomedicine and Prevention, University of Rome “Tor Vergata”, 00161 Rome, Italy; gaia.piunno@students.uniroma2.eu; 18 UOC Direzione Medica di Presidio, AST Fermo, 63900 Fermo, Italy; vincenzo.montagna@sanita.marche.it; 19 UOC Igiene degli Alimenti e Nutrizione, Dipartimento di Prevenzione, AST Macerata, 62100 Macerata, Italy; alecata@icloud.com

**Keywords:** mental health, healthcare personnel, medical residencies, longitudinal studies, protocol study

## Abstract

Medical residents constitute a vulnerable population susceptible to mental health disorders. In Italy, this was evident during the COVID-19 pandemic, when medical residents served on the front line and provided significant support to healthcare services. Therefore, the working group on “Public Mental Health” of the Medical Residents’ Council of the Italian Society of Hygiene, Preventive Medicine, and Public Health (S.It.I.) designed the “Residents’ mental health investigation, a dynamic longitudinal study in Italy” (ReMInDIt). This longitudinal study aims to assess the mental status of medical residents and to explore potential cause–effect relationships between risk/protective factors (identified among sociodemographic, residency program, and lifestyle characteristics) and mental health outcomes (anxiety and depressive symptoms). Data will be collected from a study population of 3615 residents enrolled in Italian residency programs in public health, occupational medicine, and forensic medicine through an online questionnaire that includes validated tools, requires 10 min for completion, and is disseminated by the residents’ Councils. It will be followed by a follow-up administration after 12 months. The ReMInDIt study will play a significant role in generating evidence crucial for enhancing mental health services and promoting protective factors for the mental well-being of this important segment of healthcare professionals.

## 1. Introduction

Mental health disorders pose a substantial public health hurdle. Current estimates suggest that approximately 970 million individuals worldwide grapple with such conditions, making up 13% of the global population [1,2]. The spectrum of pathological conditions affecting mental health is extensive. Depression and anxiety are the most prevalent and have the greatest impact on the global burden of disease, ranking 13th and 24th, respectively, in terms of their effect on disability-adjusted life years [3]. In particular, depression and anxiety lead to a marked decline in quality of life, resulting in psychosocial disabilities and states of distress that profoundly affect cognitive abilities, emotional regulation, and behavior [4].

Several studies highlight that healthcare professionals are characterized by a higher prevalence of symptoms typical of mental health disorders compared to the general population. For example, Gong et al.’s study reports that 25.7% of physicians exhibit depressive symptoms, and 28.1% exhibit anxiety symptoms as assessed through the Zung Self-Rating Anxiety Scale (SAS) and the Zung Self-Rating Depression Scale (SDS) [5,6,7]. The COVID-19 pandemic has exacerbated this phenomenon, causing a substantial increase in the prevalence of mental health disorders among healthcare professionals both during and after the pandemic. For instance, Hill et al.’s study reports a prevalence of depressive symptoms of 46.2% and anxiety symptoms of 45.9% [8].

Among healthcare professionals, medical residents not only represent a numerically significant portion but also serve as a fundamental resource to support health systems and constitute, at the same time, the future of the health workforce. Studies conducted before the pandemic using validated tools, such as the 21-item Depression, Anxiety, and Stress Scale (DASS-21), the General Health Questionnaire (GHQ), the Hospital Anxiety and Depression Scale (HADS-D), and the 9-item Patient Health Questionnaire (PHQ-9), had already highlighted a relevant prevalence of mental health symptoms in this category, comparable to that of other healthcare professionals [9]. The distress experienced by residents during their training period has emerged as a pervasive issue. Some studies found higher rates of depression compared to the general population [5], which have been associated with the delivery of low-quality care and an increase in errors during healthcare practices [10,11]. In this context, research also emphasizes variances among different specialties, different years of training, or in relation to gender [12,13,14]. The importance of proactively addressing this issue is evident, especially considering that residents have been found to be less prone to seek help through mental health services [15]. 

Similar to the general population, medical residents saw their personal and social lives upended by the COVID-19 pandemic and by the public policies that were enacted to prevent and control the spread of the virus. For instance, lockdown policies like the ones adopted in Italy have generally led to decreases in physical activity and increases in sedentary behavior [16]. Physical activity is well-documented as a mental health enhancer and as a protective factor against depression and anxiety [17,18]. Looking at a sample of Italian doctors, a study found that, in 2020, about 71% of them increased their food intake and 14% their alcohol consumption when compared to the pre-pandemic period [19].

The Mediterranean diet, under its wider and more complete characterization, represents a set of healthy habits related not only to food but also to physical activity and the quantity and mode of alcohol consumption. As such, it has already been linked to a number of favorable health outcomes, first and foremost in the prevention of cardiovascular disease [20]. More recently, research has explored its role in the neuropsychological domain, such as in the prevention of Alzheimer’s disease or the treatment of depression [21,22].

Medical professionals experienced all the same numerous restrictions to personal and social life as the general population, coupled with an increase in working time and professional responsibility. These stressors could have led to a change in some health behaviors, such as eating and exercising.

During the COVID-19 pandemic in Italy, medical residents from various branches of Medical Residencies played a significant role in implementing control and response measures. This required an immediate adaptation for many residents, as they were tasked with additional responsibilities beyond their usual training curriculum. Residents in the clinical services branch were particularly involved, as their training focuses on the essential skills needed to provide services crucial during a pandemic. Within the clinical services area, the class of specializations in public health includes residency programs in public health, occupational medicine, and forensic medicine [23]. These 4-year residency programs offer residents practical experiences not only in hospitals but also in local healthcare facilities, emphasizing preventive medicine and primary healthcare. During the pandemic, residents in these disciplines played a key role in supporting not only epidemiological surveillance activities, emergency healthcare management, and public health promotion at the territorial level, but also actively contributed to risk communication, contact tracing, screening, health surveillance, vaccination, and autopsy examinations. The interventions by these residents were essential in ensuring the continuity of healthcare services during the pandemic.

This resulted in a high impact of COVID-19 on the mental health of this category of professionals, with an increasing presence of depressive symptoms, as measured through questionnaires such as the PHQ-9 and the DASS-21 [24,25]. In 2022, the PHRASI study (Public Health Residents Anonymous Survey in Italy) was the first to specifically investigate Italian public health residents’ mental health [26]. Different dimensions of mental health were analyzed using previously validated tools: the Self-Related Health-5 (SRH-5) and the WHO-5 Wellbeing Index were utilized to assess wellbeing, the International Physical Activity Questionnaire (IPAQ) for physical activity, the Alcohol Use Disorders Identification Test-c (AUDITc) for alcohol abuse, the Work-Sense of Coherence Questionnaire (Work-SoC) and Work-Related Stress Questionnaire (WRSQ) for work environment, the Sick, Control, One, Fat, Food Test (SCOFF Test) for eating habits, and the Insomnia Severity Index (ISI) for sleep patterns. Additionally, the Generalized Anxiety Disorder-7 (GAD-7) was employed for anxiety assessment, and the PHQ-9 for depression. This study, with its cross-sectional design, served as a necessary and relevant first step to explore Italian medical residents’ mental health [24]. The evidence collected constitutes a solid base to move forward with the research in this area. In fact, the studies conducted so far have not pinpointed the juncture in the training path of future medical specialists where the prevalence of these mental health disorders increases. Similarly, the cause–effect relationships between possible predisposing factors and mental health outcomes have not been thoroughly investigated. A longitudinal study, one capable of tracking residents from their entry into the residency programs, can shed light on temporal associations. For these reasons, we designed the “Residents’ mental health investigation, a dynamic longitudinal study in Italy” (ReMInDIT) that sets out with the following objectives:

- Estimating the prevalence and the incidence of symptoms related to the two most prevalent mental health conditions (i.e., depression and anxiety disorders) in a sample of resident physicians attending three different types of residency programs in the class of specializations in public health (public health, occupational medicine, and forensic medicine);
- Evaluating possible risk and protective factors against the onset of these symptoms in exposed and unexposed groups, defined on the basis of socio-demographic, lifestyle, and residency-program characteristics.

## 2. Materials and Methods

ReMinDIt will be a longitudinal observational pilot study with both prospective and retrospective data collection. It will be conducted through an online questionnaire hosted on the Uniquest platform (made available by the University of Turin and based on the open-source software LimeSurvey Community Edition 3.27.22 [Copyright © 2006–2024 LimeSurvey GmbH, Hamburg, Germany]). The expected duration of the study is 18 months. Two rounds of questionnaire administrations will be conducted, with a 12-month interval between each administration:

- The first administration at baseline (Round 0) will be carried out starting 15 days after the beginning of the new academic year (15 November) and will last 1.5 months (until 30 December);
- The follow-up administration (Round 1) is expected to take place one year later with the same scheme, starting 15 days after the beginning of the new academic year (15 November) and with a duration of 1.5 months (until 30 December).

The data collected will be analyzed during the six months following the conclusion of Round 1.

### 2.1. Eligibility and Study Population

The consistency across the three residency programs in public health, occupational medicine, and forensic medicine, both in terms of duration and structure, as well as the presence of three corresponding robust societal networks facilitating effective data collection, were relevant factors for identifying the eligible population. Therefore, the inclusion criteria for participating in the ReMInDIt study will be enrollment in any course year of the residency programs of Italian Specialization Schools in public health, occupational medicine, and forensic medicine. 

The exclusion criteria will consist of having already completed the residency program, being enrolled in a residency/master program at a non-Italian university, and being enrolled in a residency program of a discipline other than those listed above.

Therefore, the study population of the ReMInDIt study is represented by all the medical residents enrolled in any of the four course years of the residency programs in public health, occupational medicine, and forensic medicine. Table 1 details the number of scholarships granted in the last four years for these residency programs [27,28,29,30]. Since enrollment in medical residencies is possible only through scholarship, the total number of scholarships funded in the last four years represents the theoretical total number of residents enrolled in the residency programs under consideration and, therefore, the number of residents eligible for this study.

### 2.2. Design

The ReMInDIt study was designed by the members of the “Public Mental Health” working group within the Medical Residents’ Assembly of the Italian Society of Hygiene, Preventive Medicine, and Public Health (S.It.I.). In the first meetings held in March 2023, the group agreed on the study design, the study population, and the recruitment pathway. In April 2023 the group started to build the questionnaires. A brainstorming session was held to list all the potential dimensions of mental health and its determinants to cover through the questionnaires’ items. 

An Excel spreadsheet (Microsoft Excel^®^ for Microsoft 365 MSO, USA, 2023) was created to gather existing tools to assess mental health and its determinants. Information to be collected included the questionnaire name, the dimension explored, the administration setting, the reference for the validation study in a non-Italian population, the reference for the validation study in the Italian population, whether the questionnaire used was paid or free, the number of items, the estimated compilation time, further references, and notes. Another spreadsheet was prepared to gather additional single questions not part of existing and validated tools. The Excel spreadsheets were filled between May and August 2023 by the working-group members based on their previous experience and thorough a literature search. 

Subsequently, the members of the working group involved both academic and non-academic experts to collect further tools and questions and obtain inputs for the elements already collected. In a meeting held in late August 2023, the group agreed on the final questions and tools to include in the questionnaires. The selection criteria were validity and reliability of the tool, brevity, possibility of self-administration, and widespread use in the literature. The selected tools and their characteristics are shown in Table 2. The number of items of each tool varied from 7 to 13, and the estimated time for completion varied from 45 s to 1 min and 30 s. All the Italian versions of the 4 selected tools were previously validated, and all of them were free of charge. In September 2023 the questionnaires were uploaded to the platform, and in October 2023 the group members accessed the questionnaires in order to perform test compilations to check the functioning of the platform and the possible presence of errors. The Uniquest platform used for the test compilation has a user-friendly interface that adapts to any type of device, thus minimizing compilation problems. The test compilations were, in fact, also essential to test the questionnaire visualization from different types of devices, in order to ensure optimal compilation in every situation.

### 2.3. Data Collection

Participation will be strictly voluntary and contingent upon the provision of explicit consent. No incentives will be offered to encourage participation. Recruitment will be conducted via a registration link disseminated through the social media channels of the national councils representing the resident doctors of the three disciplines involved: the “Medical Residents’ Council of the Italian Society of Hygiene, Preventive Medicine, and Public Health (S.It.I.)”, the “National Council for Medical Residents of Italian Society of Occupational Medicine (CoSMeL)”, and the “National Council of Young University Medical-legal Experts” [38,39,40]. The distribution of the “Round 0” link will be facilitated through mailing lists, official group chats, and conference events, supported by resident representatives from various universities.

To participate in the Round 0 (baseline) questionnaire, participants must provide an institutional email address, verifying their association with their respective universities, to uniquely identify each respondent. Participants will have the opportunity to read the informed consent for participation in the study and consent to data processing; they will access the questionnaire only after giving their consent.

The link to the Round 1 (follow-up) questionnaire will be automatically sent through the Uniquest platform to the email addresses provided. Participants will receive a personalized link via email, securing the uniqueness of their response. Medical residents will receive notifications through the communication channels activated during “Round 0”, encouraging them to check their emails.

Participants will have the flexibility to pause and continue the questionnaire at a later time of their preference. The Uniquest platform will restrict the submission to one response per participant by leveraging institutional emails and personalized links, thereby preventing multiple submissions. Answering all questions will be mandatory before submitting the questionnaire.

Participants wishing to withdraw their consent or request exclusion from Round 1 can contact the researchers at any time so that no additional data will be collected or recorded. Moreover, participants can request the removal of their registration information (name, surname, and email) and the deletion of their data before it undergoes pseudonymization or the results of the research are published.

### 2.4. Questionnaire

Soon after accessing the questionnaires of Round 0 and Round 1 through the link provided, the participants will view the information about the study and its purposes, as well as the disclosure statement regarding the processing of the participant’s personal data. Only after viewing this information, the informed consent form will be provided. 

The Round 0 questionnaire will be composed of 60 items, with an estimated completion time of about 10 min. The survey will include 4 main sections. The first will investigate sociodemographic characteristics, the second will collect data related to the residency program, the third will focus on lifestyle information, and the fourth will explore various aspects of mental health. 

In more detail, the first section, concerning socio-demographic aspects, will inquire about age, sex, weight (in kilograms), height (in meters), marital status, engagement in a stable romantic relationship, offspring, and cohabitation. It will also include questions about the region (or foreign country) of residence and internship location. Finally, it will gather information on whether the participants live in a place different from their hometown and whether they commute to reach their workplace. 

The second section will investigate aspects related to the residency program by asking the respondents about the course year and school they attend (public health, occupational medicine, or forensic medicine). This section will also include the Work-Related Stress Questionnaire (WRSQ) to investigate the working environment of the residents at baseline [33,41]. 

The third section will concentrate on lifestyles and will consist of two sub-sections. The first will analyze eating habits, physical activity, and alcohol consumption using the Chrono Med-Diet Score (CMDS) [32], a validated questionnaire consisting of 13 items, providing a score ranging from −13 to 25 points. A lower score indicates less adherence to the Mediterranean diet and a higher risk of abdominal adiposity. In the second subsection, in line with questions from the Surveillance PASSI (Progressi delle Aziende Sanitarie per la Salute in Italia) [42], smoking habits will be assessed by asking the respondent if they have smoked at least 100 cigarettes in their lifetime, if they currently smoke, and, if so, the number of cigarettes smoked per day. 

Finally, the fourth section aims to investigate different dimensions of mental health. Initially, two questions about past diagnoses of depression or anxiety disorders will be posed. Subsequently, the following instruments will be used to investigate symptoms related to anxiety and depression: the Generalized Anxiety Disorder-7 (GAD-7) [35] and the Patient Health Questionnaire 9 (PHQ-9) [37]. 

The Round 1 questionnaire will consist of 43 items, with an estimated completion time of about 8 min. The survey will include 3 main sections, omitting the investigation of lifestyles.

The first section, related to socio-demographic aspects, will ask again about weight (in kilograms) and the possibility of making ends meet with one’s own income. Additionally, with reference to the previous three months, the respondents will be asked if they have been seriously ill, experienced losses, or if any of their close family members and friends have been seriously ill. Questions will also cover the termination of a stable relationship, experiencing abuse or violence, and involvement in additional employment compatible with the residency program.

The second section will investigate detailed aspects related to the residency program, inquiring whether the respondent has undertaken two or more simultaneous traineeships in different units in the previous three months. Furthermore, using a 4-point Likert scale, the respondent will express his/her satisfaction with the school’s residency program, with particular reference to the theoretical education, the internship organization, the flexibility in carrying out traineeships in different locations belonging to the educational network of the school, the overall acquired skills, and the possibility to access courses, conferences, and research activities. Finally, the respondent will be asked to rate overall satisfaction with the attended residency program. The questions in this section are extracted and adapted from the Questionnaire of the National Observatory for Specialized Medical Training to survey the opinions of residents, which is annually administered by the Italian Ministry of University [43]. The WRSQ will be administered again to investigate changes in the characteristics of the working environment.

The third section, aiming to re-evaluate depression and anxiety symptoms, will use the same instruments from the first questionnaire. 

The list of sections, questions/questionnaires, and their possible responses with the number of items and administration timing are available in Table S1.

### 2.5. Data Analysis

The study variables will be managed as follows. Height and weight will be used to calculate the body mass index (BMI) that will be subsequently categorized according to WHO definitions (<18.5: underweight; 18.5–24.9: normal weight; 25.0–29.9: pre-obesity; 30.0–34.9: obesity class I; 35.0–39.9: obesity class II; ≥40: obesity class III) [44]. The scores deriving from the tools Chrono Med Diet, GAD-7, and PHQ-9 will be used both as continuous variables and as categorical variables based on the most commonly used cutoffs in the literature. The CMDS, ranging from −13 to 25 points, will be categorized using the cutoff of 13 as the best value to discriminate adherence to the Mediterranean diet (<13: non-adherent; ≥13: adherent) [32]. For the GAD-7 score, two categorizations will be used. The first is based on the four categories specified by the test (0–4: minimal anxiety; 5–9: mild anxiety; 10–14: moderate anxiety; and ≥15: severe anxiety), and the second is based on the cutoff identified in systematic reviews (0–7: no anxiety and ≥8: anxiety) [34,45]. For the PHQ-9 score, three categorization systems will be used. The first is based on the categories specified by the test (0–4: none–minimal; 5–9: mild; 10–14: moderate depression; 15–19: moderately severe depression; and ≥20: severe depression) [36,37], and the other two are based on a cutoff of 5 (0–4: none and ≥5: mild or more depression) and a cutoff of 10 (0–9: none-minimal and ≥10: Moderate or severe depression) as identified in the literature [46].

Dichotomous and categorical variables will be summarized using both absolute frequencies and percentages. Continuous variables will be reported as mean and standard deviation (SD) for normal distribution or median and interquartile range (IQR) for non-normal distribution. For categorical or dichotomous variables, a chi-square or Fisher’s exact test, as appropriate, will be performed to highlight statistically significant differences in distributions of the sociodemographic, lifestyle, and residency-related variables according to different levels of anxiety and depressive symptoms. Student’s t-test or the Wilcoxon–Mann–Whitney test, as appropriate, will be applied to evaluate the difference in the distribution of continuous variables among the same anxiety and depressive symptoms groups. In a longitudinal analysis, differences in anxiety and depressive symptoms between T0 and T1 will be explored using the same tests. Subsequently, two groups will be identified based on the answer to question B09 concerning overall satisfaction with the residency program. The analysis of the differences in mental health symptom variation between the two groups will be carried out using the most appropriate statistical approach depending on the variables’ distribution. Further groupings will be explored based on sociodemographic, lifestyle, and other work/training-related characteristics. Linear, polynomial, quantile, and logistic regression, depending on the nature of the variables and their distribution, will be applied. The most appropriate longitudinal analysis to identify correlates with primary outcomes will be performed for anxiety and depression symptoms, considered both as continuous and categorical/dichotomous variables. Linear mixed models (LMMs) will be used to describe the evolution of continuous variables, and generalized linear mixed models (GLMMs) will be used to analyze the longitudinal evolution of dichotomous variables.

Observations with missing data will be excluded from the analyses. Nevertheless, the structure of the questionnaire and the mandatory response enabled for each item will minimize the occurrence of missing data. The population lost at time 1 will be excluded from the longitudinal analyses but will be included in the cross-sectional analyses at time 0.

Data analysis will be performed using STATA MP 18 (StataCorp LLC., College Station, TX, USA) and R [47].

### 2.6. Sample Size

As a pilot longitudinal study, with multiple exposures that will be defined according to the distributions of the sample characteristics at Round 0, the sample-size calculation was performed using the formula by J. Charan and T. Biswas. aiming to obtain statistically significant results for both depressive and anxiety symptoms [48]. We set a precision/absolute error (d) of 5% and type 1 error $\left(Z_{1-\alpha /2}\right)$ of 5% (*p* < 0.05). Considering that the prevalence of mild-to-severe depressive symptoms was estimated by the PHRASI study at 60.1%, the resulting minimum sample size is 366 [24].

By performing the same calculations for the sample size needed to obtain statistically significant results for anxiety symptoms and using the prevalence estimate calculated from the PHRASI study, the resulting sample size is 354.

### 2.7. Ethical Considerations, Patient Information, and Written Informed Consent

The study was approved by the Ethics Committee of Umbria Region, Italy (Registration number 4617/2023, approved on 18 October 2023). Participants will be informed about the study’s objectives and data processing and will be able to download a copy of the informed-consent and data-processing agreement. The participants will express explicit consent for research enrollment and data processing. Identification of participants through their institutional email address will ensure the uniqueness of the expression of consent. Participants will be provided with an official email address dedicated to the study for any information regarding the research, data processing, and privacy protection. Through this email address, participants will have the opportunity to report any error encountered during participation. They will also have the possibility to withdraw their participation at any time of the study. Privacy protection during the analysis and interpretation of the results will be guaranteed using a blind protocol. Two main roles will be identified to ensure privacy protection. The “Data Collection Manager” will be the only researcher with access to the participants’ names and email addresses and will be responsible for the management of the platform, the collected data, and the invitation for Round 1, as well as the management of consents and requests of withdrawal from participation. The “Data Collection Manager” will perform the pseudonymization and merging of the databases from Round 0 and Round 1. Pseudonymization will be implemented using a “pseudonymization table”. The “Data Analysis Manager” will receive the pseudonymized database and will perform the analyses of the collected data. At the end of the study, the non-pseudonymized databases and the “pseudonymization table” will be deleted to further ensure privacy protection. The pseudonymized data will be stored for a maximum of 5 years.

## 3. Discussion

Mental health among medical residents unfolds as a complex and multifaceted landscape. Beyond the statistical figures, the experiences of these individuals intertwine with the intricate demands of their profession, bringing out the need for a comprehensive exploration of mental health issues in this specific cohort. This protocol establishes a rigorous methodological framework for investigating various facets of medical residents’ lives. By selecting key variables and employing specific questionnaires, our approach lays solid foundations to shed light on essential aspects related to psychological well-being within this specific population.

The socio-demographic section of the questionnaire, including parameters such as age, gender, marital status, and place of residence, allows for a better understanding of the residents’ life context. The consideration of variables, such as weight, height, and physical health status, will contribute to identifying significant correlations with mental health, thus integrating a holistic perspective.

The inclusion of existing validated tools, such as the CMDS and the WRSQ, underscores a particular attention to lifestyles and the work environment in general. These tools have been carefully selected to provide reliable measurements and allow for a comprehensive assessment of daily habits and work pressures that may influence mental health.

The section dedicated to mental health uses the GAD-7 and the PHQ-9 to investigate the most prevalent mental health conditions worldwide. These widely recognized and validated tools enable broader comparability with other studies.

Globally, healthcare professionals, including doctors undergoing residency programs, face heightened vulnerability to mental health challenges. Gong et al.’s findings, revealing substantial percentages of physicians experiencing depressive and anxious symptoms, underscore the pervasive nature of this issue within the medical community [5,6,7]. The COVID-19 pandemic has served as a magnifying lens, intensifying the pre-existing mental health burden on these professionals. Studies conducted during the pandemic, such as Hill et al.’s research, highlight the staggering prevalence of depressive and anxiety symptoms, further emphasizing the urgency of addressing mental health within the healthcare workforce [8].

In the Italian context, medical residents emerge not only as fundamental contributors to the healthcare system but also as a group facing unique stressors during their formative years of residency. Pre-pandemic studies already indicated a considerable prevalence of mental health symptoms among these professionals, aligning with global trends [9]. The challenges faced during residency training extend beyond the rigors of medical education, encompassing factors such as work-related stress, long working hours, and the responsibility associated with patient care [9]. The convergence of these challenges, exacerbated by the uncertainties brought by the pandemic, creates a complex interplay of factors that impacts the mental well-being of medical residents.

Furthermore, the proactive management of mental health concerns assumes heightened significance when considering the potential impact on the quality of healthcare delivery. Studies linking higher rates of depression to lower-quality care and an increased likelihood of errors during medical practice emphasize the interconnectedness of mental health and professional competence [10,11]. As such, initiatives aimed at fostering a supportive environment, promoting mental health awareness, and providing accessible resources gain prominence in ensuring the well-being of healthcare professionals, including those in residency programs.

The PHRASI study, conducted on a sample of Italian public health residents during the pandemic in 2022, was the first investigation to shed light on the situation, finding significant associations between variables in the training path and depressive symptoms or wellbeing in general [24,49,50]. The analyses coming from the PHRASI study found that the intention to change specialization and uncertainty about it were positively associated with depressive symptoms [24]. Conversely, willingness to work in the current internship place emerged as a protective factor against such symptoms [49]. Positive correlates, such as peer and supervisor support, increased social participation, and having a partner, were also identified, emphasizing the importance of social relationships and workplace support from the perspective of residents’ mental health [50]. These results underscore the complexity of residents’ mental health, suggesting potential areas for intervention to enhance the training experience and promote well-being.

### 3.1. Expected Results

The ReMInDIt study aims to reveal significant findings by estimating the prevalence and the incidence of symptoms related to the most prevalent mental health conditions (depression and anxiety disorders) among medical residents attending a residency program in the class of specializations in public health (public health, occupational medicine, and forensic medicine).

Previous research, including a comprehensive review and a meta-analysis, suggests that roughly 28.8% (95% CI, 25.3–32.5%) of medical residents experience depressive symptoms [9]. During the COVID-19 pandemic, anxiety was found to be particularly prevalent among healthcare professionals, with estimated rates ranging from 40% (95% CI: 29–52%) to 41.3% (95% CI: 30.2–52.9%) [51,52,53]. Similarly, depression was found to occur at a rate ranging from 30.2% (95% CI, 29.4–31.1%) to 37% (95% CI: 29–45%) among healthcare professionals [51,52,53]. Finally, Saragih et al. reported that the prevalence rate of post-traumatic stress was 49% (95% CI: 22–75%) [52]. 

Concurrently, the PHRASI study, a prior research project undertaken by the same research group as the present one, offered an overview of depression prevalence in the population under consideration for the longitudinal study. It is crucial to highlight that, during the COVID-19 pandemic, approximately 26% of Italian public health residents exhibited major symptoms related to depression [24]. We hypothesize that the longitudinal study will identify prevalence rates that are roughly comparable to those found in the aforementioned works. Specifically, we believe, to confirm the hypothesis, that depressive symptoms and other signs of impaired psychological well-being occur at specific times during residency training. Our hypothesis supports the idea that the onset of mental health symptoms among resident physicians varies throughout their training. More specifically, we estimate that factors such as increased academic and/or job-related responsibilities could contribute to the compromised state of mental well-being.

Our longitudinal methodology will allow us to effectively identify at least some critical stages or milestones in the residency program, offering valuable insights into the particular program elements that may impact mental health. Our results, unlike the preceding cross-sectional studies on the subject, should allow the formulation of new hypotheses about the causal relationships between socio-demographic, lifestyle, and residency characteristics and mental health outcomes. As an example, consistent with the PHRASI study indicating a link between work satisfaction and the emergence of depressive symptoms in medical residents [24], we expect to observe comparable trends in our investigation. Based on this research, we expect that an increase in workplace fulfillment determines a decrease in symptoms of mental health conditions, emphasizing the relevance of addressing job satisfaction as a potential protective factor.

In parallel, as reported by other studies conducted in similar populations [54,55,56], we expect that socio-demographic characteristics and lifestyle determinants other than training and working conditions may exert a substantial influence on the mental health outcomes of medical residents. A survey conducted in Nairobi, Kenya, revealed that depression was significantly linked to several factors based on a multivariate analysis [54]. These factors included reduced sleep length, high levels of perceived stress, and a lack of perceived social support [54]. Xu et al. observed a positive correlation between effective social support and a preference for a positive coping strategy [55]. Psychological resilience can be influenced by social support, which, in turn, affects coping strategies [55]. The objective of our study is to offer significant perspectives on the satisfaction of trainees, the appropriateness of the financial compensation offered through the scholarships (in relation to the place where the residency is attended), and the differences in lifestyle among the three residency programs considered.

Although aware of the potential confirmation biases that can affect, consequently, data analysis, the research group deemed it important to provide an overview of what has already been observed in the aforementioned scientific articles. Therefore, to mitigate these biases, statistical analyses will be conducted independently by at least two researchers, making use of two different data analysis programs, namely STATA and R through the RStudio graphical interface. Performing statistical analyses independently will enhance the robustness of the results and simultaneously guarantee transparency in dataset control in terms of variable processing.

### 3.2. Implications for Research, Policy, and Practice

It is worth noting that the majority of longitudinal studies investigating mental health disorders in medical residents were conducted on residents from clinical and surgical departments who differ significantly from public health, occupational medicine, and forensic medicine residents in terms of formative, work, and social life characteristics. This means that this study will contribute significantly to building evidence on mental health in a still unexplored population. 

In addition, to the best of our knowledge, this is the first study that investigates mental health in such a wide nationally representative cohort of medical residents. The insights gained from conducting this study could serve as a foundation for future research endeavors encompassing residents from diverse medical specialties, spanning both medical and surgical disciplines. Such studies could highlight potential variations in mental health statuses based on the specific area of specialization, thereby contributing to a deeper understanding of this aspect of resident well-being.

Furthermore, this study presents an opportunity to validate the Italian version of the GAD-7 among healthcare workers. While the GAD-7 is extensively employed in both research and clinical settings for identifying anxiety symptoms, its validation in Italian has thus far been limited to an inpatient population [35]. By validating the GAD-7 in a cohort of healthy young adults, we aim to furnish Italian researchers with a readily applicable and reliable assessment tool. 

Finally, among research implications, while Italian nationwide studies are lacking, single-center studies involving medical residents are present in the literature [57,58]. Our research could create a national benchmark for comparing studies on healthcare workers’ mental health that involve medical residents.

By filling a gap of knowledge in healthcare workers’ mental health, our study will allow policymakers and the directors of the residency programs under consideration to support the enhancement of psychological well-being among medical residents. Disseminating the study findings to the directors of the participating residency programs will provide them with compelling evidence regarding aspects of the program that may require adjustment to enhance residents’ psychosocial well-being, training, and working conditions. This information can serve as a catalyst for informed decision-making aimed at fostering a supportive work environment.

Moreover, the ReMInDIt study will provide evidence to support the upcoming national reform of medical residency schools and will complement the annual national survey on residency programs promoted by the National Observatory for Specialized Medical Training and give more instruments to policymakers to evaluate the quality of medical specialization schools.

Finally, not only our findings but also the considerations on the feasibility and validity of the research methods implied could serve as essential information for the implementation of a mental health monitoring program for residents across different schools and disciplines at the university and/or national level.

In conclusion, a comprehensive understanding of mental health among healthcare professionals necessitates an exploration beyond statistical metrics. By delving into the intricate intersection of professional demands, external stressors, and individual resilience, we can pave the way for targeted interventions and support systems that address the unique mental health challenges faced by these individuals. Only such holistic approaches can help in fostering a healthcare environment that not only values the well-being of its professionals but also enhances the overall quality of patient care.

### 3.3. Limitations and Strengths

Our study has some limitations. The data-collection methodology, employing self-reported questionnaires via online surveys, may introduce several biases in diverse forms. Regarding the potential technological and digital issues associated with online surveys, this specific scenario mitigates such concerns. Given that the online survey is linked to the university email of each medical resident and all Italian universities provide access to computers, technological barriers are unlikely. Furthermore, it is noteworthy that the participants are young resident doctors who are proficient with technology. While acknowledging the limited control over the respondent environment in online surveys compared to traditional methods, factors such as distractions, interruptions, or multitasking can impact response quality. To address this, we designed a relatively concise survey to maintain high respondent engagement and minimize distractions during the questionnaire. The online-survey approach helps mitigate biases, including social desirability bias, which involves the tendency of study participants to conform as much as possible to what is socially acceptable out of fear of being recognized, or the self-serving bias, which implies presenting themselves in a favorable light. In this context, the guarantee of personal data protection of the questionnaire should help reduce these biases, although the fear of recognition may have repercussions on data collection. Recall bias manifests as a systematic error when participants inaccurately recall past events or experiences, either by remembering them incorrectly or omitting important details. To mitigate the impact of recall bias, we opted to narrow as much as we could the timeframe for questions related to past information. Misclassification bias could also occur if individuals are assigned to a category different from the one they should belong to, for instance assigning a resident into different exposure or outcome groups, leading to inaccurate estimates of the association between variables. This will be limited by adopting cutoffs for the selected measurement scales that are already validated and widely used in the literature, as described in the previous chapters.

The timing of the baseline administration of the questionnaire (Round 0) could introduce biases in the results for the residents of the first course year. In fact, by conducting the questionnaire shortly after the start of the residency program, we may miss the initial phase in which the incidence of anxiety and depression symptoms starts to increase. This would result in an underestimation of the effect size that certain training-related variables have on mental health. However, recruiting medical residents after the beginning of the residency training is dictated by necessity. As a matter of fact, the recruitment networks (“Medical Residents’ Council of the Italian Society of Hygiene, Preventive Medicine, and Public Health (S.It.I.)”, the “National Council for Medical Residents of Italian Society of Occupational Medicine (CoSMeL)”, and the “National Assembly of Young University Forensic Doctors”) do not have the ability to get in contact with residents before they enter the program. Taking into account all these aspects, we choose to start the data collection 15 days after the beginning of the training courses. This timeframe provides first-year residents with the opportunity to acclimate to their new training environment, while also allowing them to become acquainted with the three Councils involved in the recruitment process. This fosters wider participation in the subsequent enrollment phase. It must be noted that this phenomenon is limited to first-year residents, and it has only a partial impact on the estimated incidence rates, which will be calculated across the entire sample that includes residents from all four course years.

The voluntary nature of the participation in the study may introduce a selection bias, as it is possible that only residents who are more active in their respective medical residents’ networks or those more interested in the subject may be willing to participate in the survey. On the other hand, it is important to also consider that residents with mental health-related symptoms, such as depressive symptoms, may be less motivated to take part in the study or complete the follow-up survey, leading to a potential underestimation of the incidence rate. However, it is crucial to take into account that free and voluntary participation by subjects interested in being involved in the study may have positive long-term effects, reducing the loss of participants in the follow-up. The lost-to-follow-up bias will be further limited by informing participants about the timing of the administration of the follow-up questionnaire from their enrollment. Additionally, the official communication channels of the medical residents’ networks will be used to announce and spread the opening of each round of data collection.

Despite these limitations, the study has some strengths to be considered. As outlined above, this will be the first research to put in place a longitudinal investigation of mental health in a nationally representative sample of medical residents. The questionnaires will contain reliable, free, and widely used tools already validated in Italian. In particular, the Work-Related Stress Questionnaire was specifically validated in a sample of Italian medical residents. The selection of these tools was based also on their estimated completion time, which is relatively short. Employing a professional platform for data-collection management ensures the mitigation of errors during this phase. The automated email invitations for completing the Round 1 questionnaire will ensure the smooth continuation of the follow-up process. Moreover, supplementing the initiation of the follow-up phase with notifications via multiple communication channels will help decrease attrition rates. Enabling the mandatory completion option for all questionnaire items will help prevent the occurrence of missing data. The implementation of a blind protocol in the phase of data analysis will be crucial for ensuring privacy protection and enhancing participants’ trust in the handling of personal data. This measure is expected to positively impact the response rate. The Italian national councils representing the resident doctors maintain a comprehensive national network, facilitating their ability to reach residents across all Italian universities. This will ensure the extensive distribution of the invitations for Round 0. Finally, this study builds upon the evidence gathered from the PHRASI study. This pilot cross-sectional investigation was the first to explore mental health among medical residents. The insights gained from that experience facilitated the identification of optimal assessment tools for measuring mental health symptoms and their determinants, as well as refining the methodology of the ReMInDIt study to ensure both methodological rigor and feasibility.

## 4. Conclusions

The ReMInDIt is the first study with the aim of estimating both the prevalence and the incidence of symptoms linked to mental health among Italian residents enrolled in public health, occupational medicine, and forensic medicine residency programs. Thanks to its longitudinal design, this study represents a starting point for monitoring the mental health of the target population over time. The evidence gathered will serve as a guiding resource for universities, residency-program directors, and policymakers, aiding them in enhancing the quality of training and working conditions for medical residents. Implementing mental health interventions based on the data coming from the ReMInDIt study will be possible thanks to the several tools and techniques that fall under the mental health and psychosocial support instruments. The feasibility and validity of the application of these techniques on the specific population of medical residents represent a still unexplored area of study. Furthermore, this research will make a valuable contribution to the international discourse on healthcare workers’ mental health, which stands as a crucial pillar for the sustainability of healthcare systems globally and for the overall quality of patient care. 

## Figures and Tables

**Table 1:** Detail of the total and per-year number of scholarships funded for public health, occupational medicine, and forensic medicine residency programs.

	Scholarships Funded				
Residency program	2020	2021	2022	2023	TOTAL
Public Health	575	780	542	630	2509
Occupational Medicine	188	236	221	227	872
Forensic medicine	158	200	171	195	724
Total	921	1216	934	1052	4105

Nevertheless, it is necessary to consider both non-assigned and dropped scholarships. According to the latest statistics available, these amount to 14.5% of the total for the residency program in public health, 9.5% for occupational medicine, and 6% for forensic medicine, yielding an estimate of approximately 490 lost scholarships [31]. Therefore, the estimated total number of residents currently enrolled in the three residency programs under consideration is 3615.

**Table 2:** Characteristics of the selected tools to include in the final questionnaire.

Name of the Questionnaire	Original Validation Article	Validation for Italian Population	Free of Charge	N. of Items	Estimated Time of Compilation (Minutes)	Aim	Further References and Notes
Chrono Med Diet Score	De Matteis C et al., 2023 [32]	De Matteis C et al., 2023 [32]	Yes	13	1:30	Measuring adherence to Mediterranean Diet	Italian version available at https://www.chronomeddiet.org/ (accessed on 17 February 2024)
Work-Related Stress Questionnaire	Cedrone F et al., 2024 [33]	Cedrone F et al., 2024 [33]	Yes	13	1:30	Description of working environment	Pre-pilot study at https://pubmed.ncbi.nlm.nih.gov/32614365/ (accessed on 17 February 2024)
General Anxiety Disorder-7	Spitzer RL et al., 2006 [34]	Bolgeo et al., 2023 [35]	Yes	7	0:45	Screening for generalized anxiety disorder	Italian version available at https://www.ecfs.eu/sites/default/files/general-content-files/working-groups/Mental%20Health/GAD7_Italian%20for%20Italy.pdf (accessed on 17 February 2024)
Patient health Questionnaire-9	Kroenke K et al., 2001 [36]	Mazzotti E et al., 2003 [37]	Yes	9	1:00	Screening of depression	Italian version available at https://www.demenzemedicinagenerale.net/images/test/PHQ-9_Ok_20-2-2016.pdf (accessed on 17 February 2024)

## Data Availability

Data are contained within the article and Supplementary Materials.

## Supplementary Materials

The following supporting information can be downloaded at https://www.mdpi.com/article/10.3390/healthcare12101020/s1, Table S1: Structure and questions included in the questionnaires.
