# Supplementary material for: Study Protocol for the Residents’ Mental Health Investigation, a Dynamic Longitudinal Study in Italy (ReMInDIt)
Source: Healthcare (Basel). 2024 May 15;12(10):1020. doi: 10.3390/healthcare12101020 (PMC11121525; doi:10.3390/healthcare12101020)
Supplement: Supplementary file 1 [file healthcare-12-01020-s001.zip › healthcare-2989348-supplementary.pdf]

Supplementary material

Table S1. Structure and questions included in the questionnaires.

Sections		Questions			Items	Round	
**Cod.**	**Description**	**Cod.**	**Description**	**Answer**		**0**	**1**
A	Sociodemographic characteristics	A01	Sesso	(M; F)	1	x	
A	Sociodemographic characteristics	A02	Età	(Anni)	1	x	
A	Sociodemographic characteristics	A03	Peso	(kg)	1	x	x
A	Sociodemographic characteristics	A04	Altezza	(m)	1	x	
A	Sociodemographic characteristics	A05	Stato civile	(Coniugato/a; libero/a)	1	x	
A	Sociodemographic characteristics	A06	Sei in una relazione sentimentale stabile?	(Sì, no)	1	x	
A	Sociodemographic characteristics	A07	Hai figli?	(Più di 2, uno, zero)	1	x	
A	Sociodemographic characteristics	A08	Abiti con altre persone?	(Sì, no)	1	x	
A	Sociodemographic characteristics	A09	Regione di residenza	(Elenco regioni italiane + estero)	1	x	
A	Sociodemographic characteristics	A10	Regione di tirocinio/lavoro	(Elenco regioni italiane + estero)	1	x	
A	Sociodemographic characteristics	A11	Rispetto alla tua residenza, sei fuori sede?	(Sì, no)	1	x	
A	Sociodemographic characteristics	A12	Rispetto al luogo in cui abiti, sei pendolare?	(Sì, no)	1	x	
A	Sociodemographic characteristics	A13	Durante i tre mesi scorsi: sei stato gravemente malato?	(Sì; no)	1		x
A	Sociodemographic characteristics	A14	Durante i tre mesi scorsi: hai avuto lutti o qualcuno tra i tuoi familiari e amici più stretti è stato gravemente malato?	(Sì, no)	1		x
A	Sociodemographic characteristics	A15	Durante i tre mesi scorsi: hai interrotto una relazione stabile?	(Sì, no)	1		x
A	Sociodemographic characteristics	A16	Durante i tre mesi scorsi: sei stato vittima di abusi o di violenza?	(Sì, no)	1		x
A	Sociodemographic characteristics	A17	Durante i tre mesi scorsi hai svolto lavori compatibili con la scuola di specializzazione?	(Sì, con ore da contratto svolte dentro l'orario formativo; Sì, con ore da contratto svolte extra orario formativo; no)	1		x
A	Sociodemographic characteristics	A18	Come riesci economicamente ad arrivare a fine mese?	(Facilmente, Con aiuto di altri, Con difficoltà)	1		x
B	Residency program	B01	Anno di specializzazione	(1°; 2°; 3°; 4°)	1	x	
B	Residency program	B02	Quale scuola di specializzazione frequenti?	(Igiene e medicina preventiva; Medicina del Lavoro; Medicina Legale; altro: specificare)	1	x	
B	Residency program	B03	Durante i tre mesi scorsi hai svolto tirocini paralleli in servizi/unità operative differenti? (Es. 2	(Sì; no)	1		x

			giorni/sett. in un servizio e 3 giorni/sett. in un altro servizio)				
B	Residency program	B04	La tua Scuola ti ha offerto un'attività didattica formale in linea con il piano formativo?	(Assolutamente sì, più sì che no, più no che sì, assolutamente no)	1		x
B	Residency program	B05	L'organizzazione delle attività didattiche e dei tirocini è abbastanza accurata da evitarne la sovrapposizione?	(Assolutamente sì, più sì che no, più no che sì, assolutamente no)	1		x
B	Residency program	B06	Sei soddisfatto/a della possibilità di ruotare sulle sedi della rete formativa?	(Assolutamente sì, più sì che no, più no che sì, assolutamente no)	1		x
B	Residency program	B07	Ritieni che la preparazione fino ad oggi ricevuta nel corso della specializzazione sia adeguata allo svolgimento della tua futura attività di specialista?	(Assolutamente sì, più sì che no, più no che sì, assolutamente no)	1		x
B	Residency program	B08	Nella tua Scuola gli specializzandi hanno le stesse possibilità di accedere a corsi, congressi, collaborazione alle attività di ricerca?	(assolutamente sì, più sì che no, più no che sì, assolutamente no)	1		x
B	Residency program	B09	Ti ritieni complessivamente soddisfatto/a della Scuola di specializzazione?	(Assolutamente sì, più sì che no, più no che sì, assolutamente no)	1		x
B	Residency program	B10-22	Work-related Stress Questionnaire	(Mai; raramente; qualche volta; spesso; sempre)	13	x	x
C	Lifestyle information	C01-13	Chrono Med Diet Score	/	13	x	
C	Lifestyle information	C14	In tutta la tua vita, hai fumato in tutto almeno 100 sigarette (5 pacchetti da 20 sigarette)?	(Sì, No, non so o non ricordo)	1	x	
C	Lifestyle information	C15	Attualmente fumi sigarette?	(Sì, no)	1	x	
D	Mental health	D01	Hai mai ricevuto una diagnosi di depressione?	(Sì, no)	1	x	
D	Mental health	D02	Hai mai ricevuto una diagnosi di disturbo dello spettro ansioso?	(Sì, no)	1	x	
D	Mental health	D03-09	Sintomi ansiosi: GAD-7	(Mai; alcuni giorni; per oltre la metà dei giorni; quasi ogni giorno)	7	x	x
D	Mental health	D10-18	Sintomi depressivi: PHQ-9	(Mai; alcuni giorni; per oltre la metà dei giorni; quasi ogni giorno)	9	x	x
				**Total items**		**60**	**43**

*The table displays the structure of the questionnaires at Round 0 and Round 1. The columns, in order, feature: the sections' code and descriptions, the questions' codes and text, possible answers, the items count, and whether the question was included in Round 0, Round 1, or both rounds. Questions extracted from validated questionnaires are not fully transcribed.
